# Epidermodysplasia Verruciformis and Vδ2 γδ T-cell Expansion in STK4 Deficiency

**DOI:** 10.1007/s10875-024-01780-z

**Published:** 2024-08-07

**Authors:** Wenjing Ying, Xin Long, Travis Vandergriff, Hemanth Karnati, Meghan Heberton, Mingyi Chen, Xiaochuan Wang, Christian Wysocki, Xiao-Fei Kong

**Affiliations:** 1 Division of Digestive and Liver Diseases, Department of Internal Medicine, UT Southwestern Medical Center, 5323 Harry Hines Blvd, Suite J5.136, Dallas, TX 75390-9151 USA; 2 Department of Clinical Immunology, Children’s Hospital of Fudan University, National Children’s Medical Center, Shanghai, 201102 China; 3 Hepatic Surgery Center, Tongji Hospital, Tongji Medical College, Huazhong University of Science and Technology, Wuhan, 430030 China; 4 Departments of Dermatology and Pathology, UT Southwestern Medical Center, Dallas, TX 75390 USA; 5 Department of Pathology, Harold C. Simmons Comprehensive Cancer Center, UT Southwestern Medical Center, Dallas, TX 75390 USA; 6 Division of Allergy and Immunology, Department of Internal Medicine, UT Southwestern Medical Center, Dallas, TX 75390 USA; 7 McDermott Center for Human Growth and Development, UT Southwestern Medical Center, 5323 Harry Hines Blvd, Suite J5.136, Dallas, TX 75390-9151 USA; 8 Department of Dermatology and Dermatopathology, Harold C. Simmons Comprehensive Cancer Center, UT Southwestern Medical Center, Dallas, TX 75390 USA

## Abstract

The clinical penetrance of infectious diseases varies considerably among patients with inborn errors of immunity (IEI), even for identical genetic defects. This variability is influenced by pathogen exposure, healthcare access and host-environment interactions. We describe here a patient in his thirties who presented with epidermodysplasia verruciformis (EV) due to infection with a weakly virulent beta-papillomavirus (HPV38) and CD4^+^ T-cell lymphopenia. The patient was born to consanguineous parents living in the United States. Exome sequencing identified a previously unknown biallelic *STK4* stop-gain mutation (p.Trp425X). The patient had no relevant history of infectious disease during childhood other than mild wart-like lesion on the skin, but he developed diffuse large B-cell lymphoma (DLBCL) and EBV viremia with a low viral load in his thirties. Despite his low CD4^+^ T-cell count, the patient had normal counts of CD3^+^ cells, predominantly double-negative T cells (67.4%), which turned out to be Vδ2^+^ γδ T cells. γδ T-cell expansion has frequently been observed in the 33 reported cases with STK4 deficiency. The Vδ2 γδ T cells of this STK4-deficient patient are mostly CD45RA^-^CD27^+^CCR7^+^ central memory γδT cells, and their ability to proliferate in response to T-cell activation was impaired, as was that of CD4^+^ T cells. In conclusion, γδ T-cell expansion may act as a compensatory mechanism to combat viral infection, providing immune protection in immunocompromised individuals.

**Supplementary Information:**

The online version contains supplementary material available at 10.1007/s10875-024-01780-z.

## Introduction

Epidermodysplasia verruciformis (EV) (OMIM 226400) is a rare autosomal recessive skin disorder characterized by persistent verrucae and pityriasis versicolor-like or warty cutaneous lesions due to impaired defenses against beta-papillomaviruses (β-HPVs). This impairment increases the risk of non-melanoma skin cancers, such as Bowen disease and squamous cell carcinoma [1]. β-HPVs are ubiquitously present in the general population but do not generally cause disease; however, at least 18 types of β-HPV have been reported to cause EV in rare cases [2]. Initial observations of EV in consanguinity families lead to the hypothesis that EV might be a monogenic disease. The discovery of the principal causal genes for EV, *EVER1* and *EVER2*, by the late Gerard Orth led to the conclusion that patients with EV had an impaired keratinocyte-intrinsic immune response to β-HPVs [3]. These two genetic disorders account for over 50% of cases of EV in patients with no particular susceptibility to viral or bacterial infections, a condition known as “typical EV”. Further studies identified 24 EV patients homozygous for deleterious variants of the *CIB1* gene, encoding the CIB1 protein of the CIB1-EVER1-EVER2 complex involved in the lifecycle of β-HPVs [4]. Patients with typical EV develop skin lesions early in childhood, and 30–70% of EV patients subsequently develop squamous cell carcinoma, particularly those with HPV5 and HPV8 infections [5–7]. By contrast, atypical EV is the condition identified in patients with other inborn errors of immunity (IEI) in addition to cutaneous β-HPV infection. Several patients with IEI, including AR *RHOH*, *LCK*,* DOCK8* and *STK4* deficiencies, have been reported to have histological findings consistent with EV due to β-HPVs [8]. All these patients had CD4^+^ T-cell lymphopenia, but not all patients with T-cell deficiency develop EV; by contrast, penetrance is complete in patients with typical EV and AR *CIB1*-*EVER1*-*EVER2* deficiency [1].

We report here a case in his thirties who presented at our immunology clinic with EV and T-cell lymphopenia. This patient was found to be homozygous for a previously unknown nonsense variant of *STK4*. Human STK4 (serine/threonine kinase 4) deficiency was first described in 2012 as an autosomal recessive inborn error of immunity characterized by recurrent bacterial, viral and fungal infections due to profound CD4^+^ T lymphopenia in the first decade of life [9, 10]. STK4 is a highly conserved HIPPO pathway phosphorylated forkhead homeobox type O (FOXO) transcription factor controlling cell growth, tumorigenesis, and apoptosis. We characterized the clinical and immunological features of this patient diagnosed in his thirties with a condition typically identified early in life.

## Methods

### Clinical Study and Sequencing

Genomic DNA (gDNA) was isolated from the peripheral blood with the Quick-DNA™ Miniprep Plus Kit (ZYMO research, USA). Whole Exome Sequences (WES) were obtained at GENEWIZ with a Twist Human Comprehensive Exome kit and next-generation sequencing was performed on a NovaSeq XPlus sequencer. We followed the GATK best-practice workflow to establish our pipeline for variant calling [11]. Briefly, raw-data quality metrics were calculated with FastQC and sequencing reads were mapped onto the reference genome (GRCh38) with BWA. PICARD and GATK4 were used to perform local realignment and base-quality recalibration, respectively, to ensure high-quality base calls. The variant call format (VCF) file was annotated with VEP. Genetic diagnosis was facilitated by using a gene list for IEIs developed by the Expert Committee of the International Union of Immunological Societies (IUIS). β-HPVs were amplified from DNA extracted from the lesion swab, as previously described [12]. Sanger sequencing was used to confirm the genotypes of β-HPVs and the *STK4* variant. This study was approved by the institutional review boards of UTSW.

### Immunophenotyping and T-cell Proliferation

Immunophenotyping was performed as previously described but with several modifications [13]. First, freshly thawed PBMCs (3–5 × 10^6^ cells) were simultaneously incubated with LIVE/DEAD Fixable Blue dye and FcR blocking reagent on ice for 15 min. in the cells were washed and then incubated on ice for 30 min in Brilliant Stain Buffer Plus with the antibodies against the following: CD45RA, CD16, CD14, CD194(CCR4), Integrin β7, CD56, CD123, CD38, IgD, PD1, HLA-DR, CD69, CD183 (CXCR3), CD196 (CCR6), Vδ2 TCR, CCR7, CD141, CD8, CD3, CD11b, TCR-γ/δ, TCR-Vδ1, CD4, CD27, CD24, CD19, CD25, CD11c, CRTH2, CD127(IL-7R), CD1c, TCR-α/β, CD161, CD185 (CXCR5), and CD57. For intracellular perforin staining, cells were fixed by incubation with 4% paraformaldehyde for 10 min at 37 °C and then stained in 0.1% saponin and 1% BSA in PBS. Cell acquisitions were performed on a Cytek Aurora cytometer and data were analyzed with FlowJo Version 10 software. Details of the antibodies used are provided in Table S1. A carboxyfluorescein succinimidyl ester (C-FSE) proliferation assay was performed to assess T-cell proliferation. PBMCs were stained with 1 µM C-FSE (C34554, Thermo Fisher) at 37 °C for 20 min, washed three times, and then used to seed a U-bottom 96-well plate at a density of 1 × 10^6^ cells/ml. The concentration of IL-2 (31058, Cell Signaling) in the medium was maintained at 50 ng/ml and cells were incubated with or without mitogens, including ImmunoCult™ Human CD3/CD28/CD2 T cell Activator (10970, STEMCELL) and phytohemagglutinin-M (PHA-M, Roche). After incubation for five days, the cell were incubated with anti-CD4-PE (300508, Biolegend) and anti-Vd2 TCR-BV750 (B6 clone, 747204, BD) antibodies, together with Aqua Live/Dead stain (L34966, Thermo Fisher), and analyzed on an LSRII (BD Biosciences).

### Statistical Analysis

All statistical analyses were performed in Prism 8. The statistical significance of quantitative differences between groups was assessed in unpaired *t* tests. *P* values below 0.05 were considered statistically significant. t-SNE map was used as a visualization tool for the qualitative assessment of cell population diversities for both the patient and a healthy control. The impact of limiting the markers used to construct a global t-SNE map to general lineage markers only was assessed by running t-SNE with the following markers alone: CD45RA, CD3, CD4, CD8, vδ2TCR, CD19, CD27, CD14, CD11c, CD56 and CD16.

## Results

### Clinical Manifestations and Histological Characteristics of the Proband

**Fig. 1:**
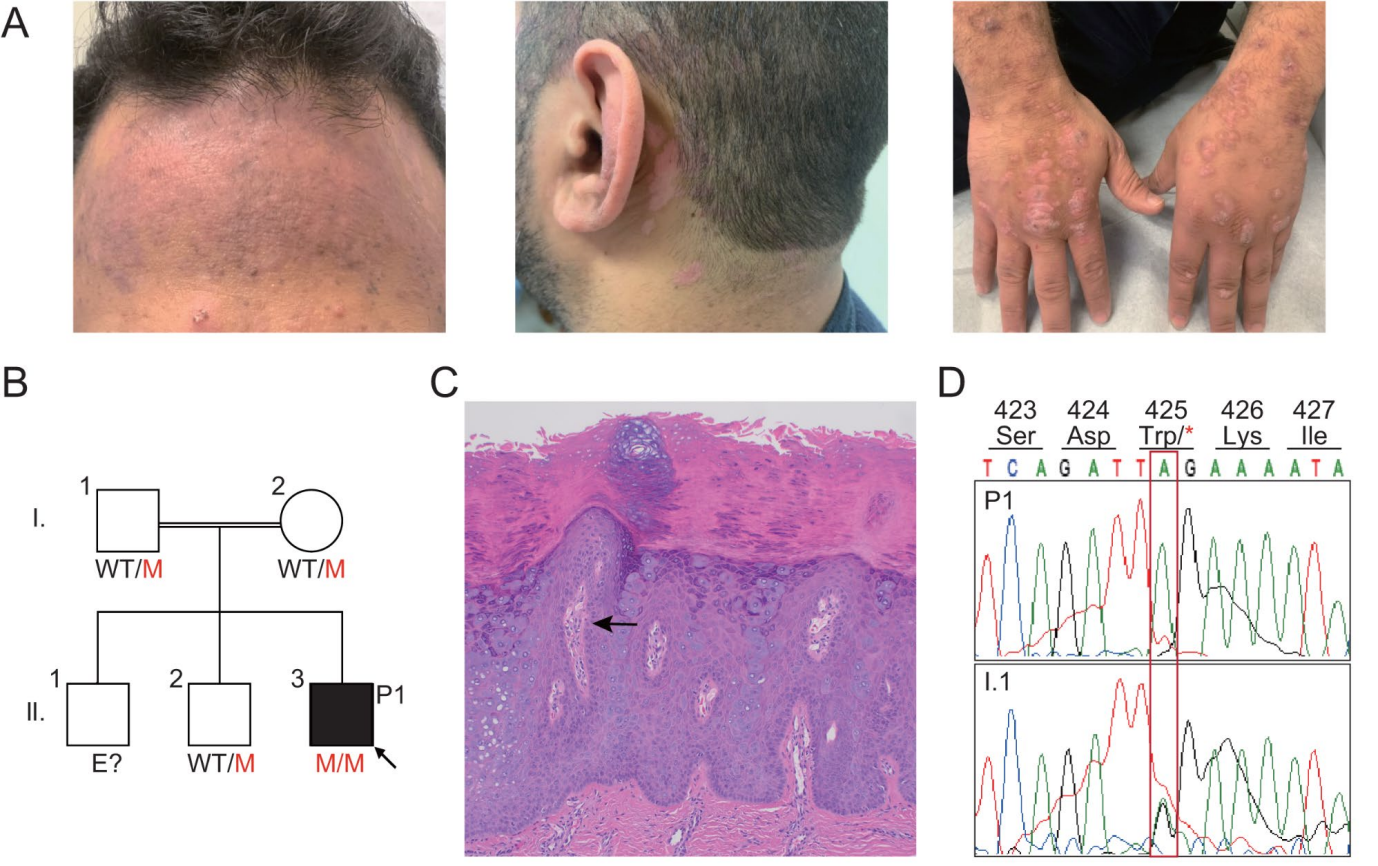
An STK4-deficient patient with HPV38-associated EV, atopic dermatitis and lymphoma. (**A**) Skin manifestations of scaly plaques and pigmentation on the forehead, and scaly erythematous plaques behind the ears, on the back of the hand and on the back of both wrists; (**B**) Family pedigree and variant segregation; (**C**) H&E staining of an EV lesion (×200 magnification); Numerous enlarged keratinocytes with steel gray cytoplasm are present (Arrowed). (**D**) Sanger sequencing confirmed the presence of the *STK4* p.Trp425X variant in the homozygous state in P1 and the heterozygous state in his father

The proband is a 31-year-old man living in Texas, the United States, who was born to consanguineous parents who had emigrated from the Middle East. He was referred to the immunology clinic for T-cell lymphopenia. He had no significant family history of immunodeficiency and first presented with flat warts on his hands at the age of three years. These warts subsequently spread diffusely over the scalp, ears, and upper extremities (Fig. 1A). The patient had normal growth and development, with no pneumonia, oral infections, or abscesses, and no significant history of prior infections other than pericarditis at the age of 12 years, and mild cellulitis not requiring drainage or antibiotics in his twenties. The proband is the only person from his family affected (Fig. 1B). At the age of 29 years, a mass causing sore throat and hoarseness was detected in the epiglottis. Biopsy and pathology analysis revealed a diffuse large B-cell lymphoma (DLBCL), with positive staining for CD20, CD79a, BCL2 and BLC6, but negative for CD10; 30% of the cells were positive for MIB-1 but MUM1 (IRF4) staining was negative, suggesting a germinal center-type lymphoma. The patient received four cycles of R-CHOP chemotherapy for stage IIa disease and complete remission was achieved. A PET-CT scan at two years of follow-up revealed an intensely hypermetabolic lung nodule in the left hilum. Biopsy results indicated an inflammatory process, suggesting a contained infection; no pathogens were identified.

Physical examination was unremarkable for the neck, lungs, heart, and abdomen. There was no lymphadenomegaly or hepatosplenomegaly. The patient had hypopigmented papules on his skin, flattened erythematous papules on the trunk that coalesced into plaques with a cobblestone pattern, and erythematous scaly plaques, mostly on the back of the hands and both wrists and behind the ears. He had scaly plaques on his scalp, upper arms, and forehead. Biopsy of the lesions on the back of the left wrist revealed acanthosis and papillomatosis, with numerous enlarged keratinocytes with a steel-gray cytoplasm, consistent with epidermodysplasia verruciformis (EV) (Fig. 1C). Amplification results for the DNA from the skin lesion swab revealed the presence of a β-HPV, which Sanger sequencing identified as HPV38, one of the β-HPVs known to cause EV [1] (Fig. S1). In addition, the EBV serology tests were positive for EBV-VCA and EBV-EA-IgG, together with EBV-DNA at 3.28 log IU/ml. EBV mRNA transcripts (*EBNA2*, *BZLF1* and *gp330/220*) were undetectable in the RNA isolated from PBMCs (data now shown), suggesting a chronic latent EBV infection.

### Characterization of the Genetic and Immunological Features

WES revealed that the proband was homozygous for a stop-gain variant of the *STK4* gene (NM_006282.5:c.1274G > A, p.Trp425X, Fig. S2A and S2B), which was confirmed by Sanger sequencing (Fig. 1D). Both his parents and one of his siblings were heterozygous for the variant. This variant is ultra-rare, absent from the gnomAD and “*All of US*” databases, predicted to be deleterious by SIFT, PolyPhen with a CADD score of 43, and classified as PVS1 [14]. Laboratory tests showed the patient’s hemoglobin levels and platelet counts to be normal, whereas he presented profound lymphopenia (total leukocyte count, 4.7 × 10^6^/L; lymphocytes 0.7 × 10^6^/L; neutrophils 3.3 × 10^6^/L). The patient had low CD4^+^ T-cell counts (136–292 cells/µl), normal B and NK cell proportions, a slightly high percentage of CD8^+^ T cells and a low CD4^+^ to CD8^+^ T-cell ratio (CD4/CD8 = 0.33–0.85; normal = 0.86-5). Immunoglobulin (Ig) profiling revealed low levels of IgM, with normal IgG and IgA levels (Table S2).

**Fig. 2:**
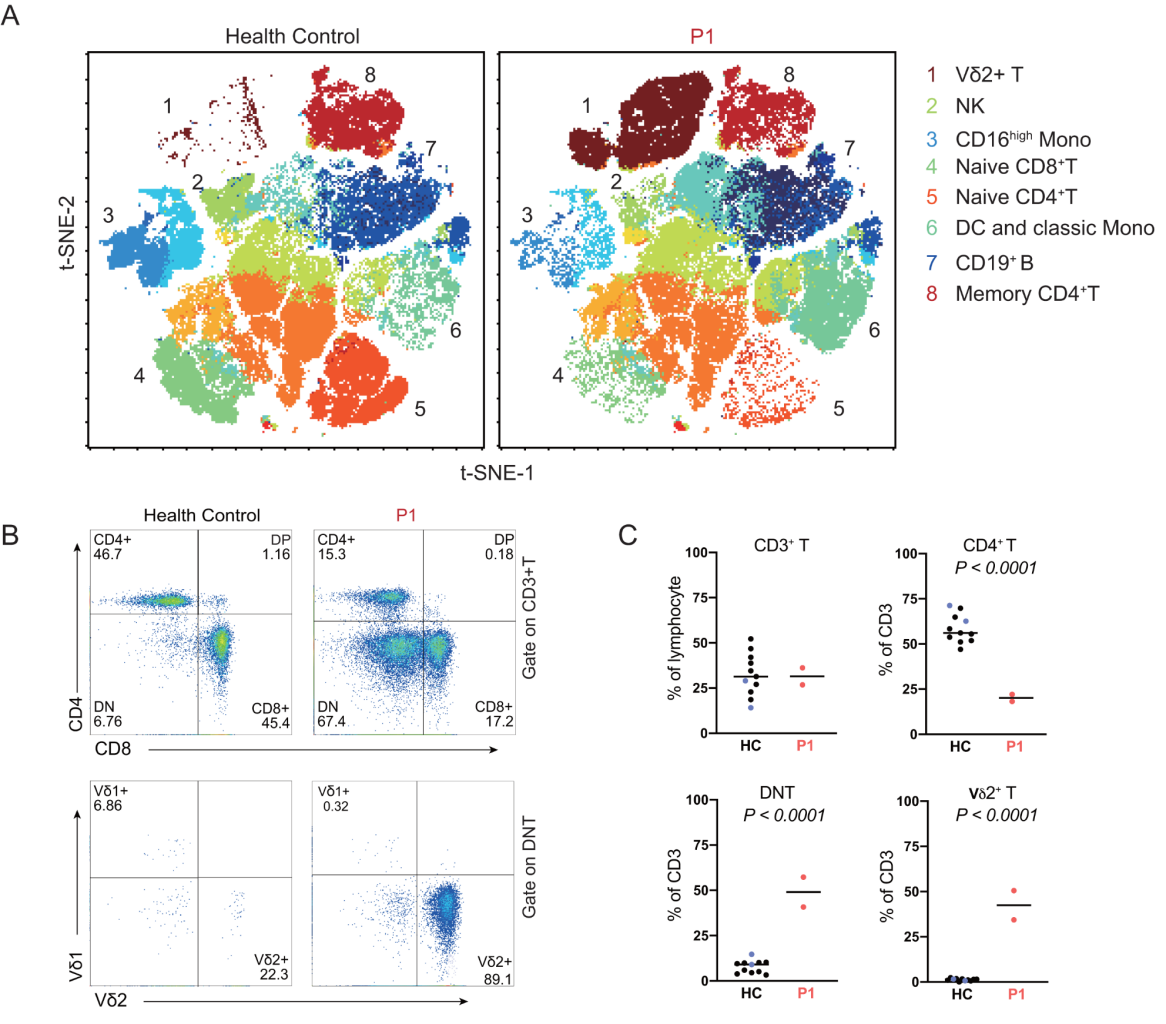
Immunophenotyping revealed low CD4^+^ T-cell levels, normal CD3^+^ T-cell levels and a high level of double-negative T cells in the proband. (**A**) t-SNE results for PBMCs, showing monocytes, NK, B, CD4^+^ and Vδ2 T cells; (**B**) T-cell subsets and expansion of the Vδ2 T-cell subset in the patient. (**C**) Percentages of lymphocyte subsets within the CD3^+^ population of the patient (red), his parents (blue) and healthy controls (black). Two indepenant experiments were performed for the patient

We then investigated the immunological impact of this variant by performing comprehensive immunophenotyping (Fig. 2A), which revealed low levels of CD4^+^ T cells in the patient, particularly for naïve CD4^+^ and CD8^+^ T cells (Fig. 2A and Fig. S2C), consistent with previous reports (Table S3) [9, 10, 15–28]. Interestingly, the patient had a significantly higher than normal proportion of double-negative T cells (DN 67.4% among CD3^+^ T cells, Fig. 2B). Most of these DN T cells were CD3^+^ Vδ2^+^ γδ T cells, which accounted for 89.1% of all DN T cells (Fig. 2B). CD4^+^ T lymphopenia and Vδ2^+^ T-cell expansion were not transient in the proband; these phenotypes were reproducible in blood tests performed a few months apart (Fig. 2C). Expansion of the γδ T-cell population is frequently reported in IEI [29], but expansion of the Vδ2 T-cell subset is a particularly interesting phenotype in the context of STK4 deficiency.

### Investigating the Immunological Profile of Vδ2 T Cells in STK4 Deficiency

**Fig. 3:**
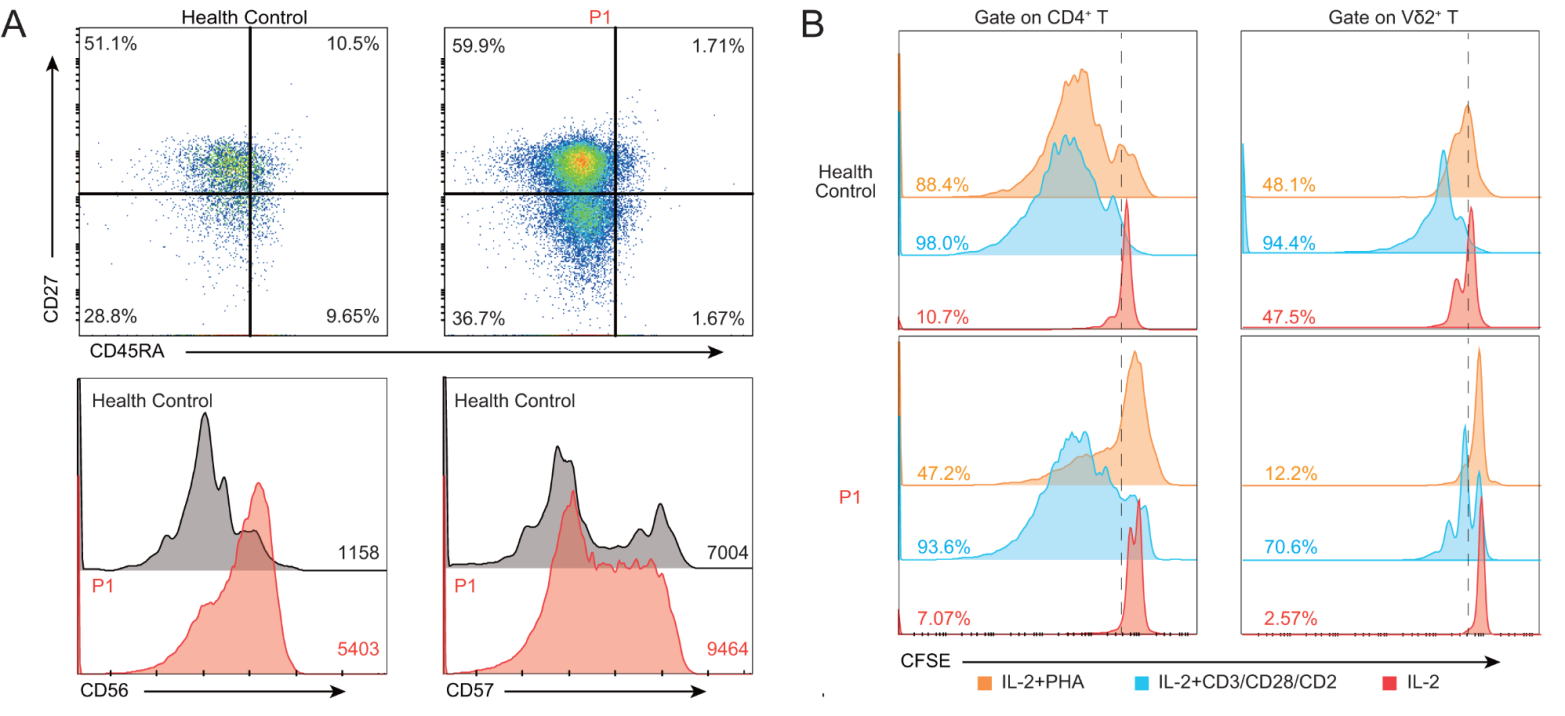
Vδ2 T cells from the STK4-deficient patient, displaying features of both naïve and effector cells, high levels of CD56 and CD57 expression, and mildly impaired proliferation upon T-cell activation. (**A**) CD27^+^CD45RA^-^ T cells were the major subset of Vδ2 T cells. Histogram of CD56 and CD57 expression on Vδ2 T cells. (**B**) CFSE proliferation assay with CD4^+^ T T cells and Vδ2^+^ T cells incubated with 50 ng/ml IL-2 plus 250 ng/ml PHA or 1:100 T cells anti-CD3/CD28/CD2 T-cell activator

An expansion of γδ T cells has been observed in five patients with STK4 deficiency with or without EV [9, 26, 28]. In humans, Vδ2 T cells are the predominant subset in the γδ T-cell population of peripheral blood; they respond to prenyl pyrophosphate metabolites (P-Ags) and have been implicated in antiviral and antitumor immunity [30]. Most of the Vδ2 T cells from the proband were CD27^+^ CD45RA^−^, also exhibiting high CCR7 expression (Fig. S3), consistent with an innate-like Vγ9^+^Vδ2^+^ central memory T-cell phenotype [31–33], but a minority had the CD27^lo^ CD45RA^+^ effector phenotype (Fig. 3A). Vδ2 T cells from the proband displayed remarkably high levels of CD57 and CD56 expression (Fig. 3A), but did not express PD-1 or perforin (Fig. S2D). CD56 and CD57 are markers of exhausted γδ T cells. We therefore investigated the proliferative capacity of the cells. Proliferation rates in response to IL-2 with either PHA or CD3/CD28/CD2 stimulation were higher for CD4^+^ T cells than for Vδ2^+^ T cells. CD3/CD28/CD2 stimulation induced a more robust T-cell proliferation than PHA. The proband had low rates of CD4^+^ T-cell proliferation in response to both CD3/CD28/CD2 and PHA, but this impairment of proliferation was more marked for PHA stimulation (Fig. 3B), consistent with previous reports [9, 16, 18, 24, 26–28]. Vδ2^+^ T cells from the proband also displayed an impairment of proliferation relative to the healthy control. In conclusion, the cells of the expanded Vδ2 T-cell population from this STK4*-*deficient patient had mostly innate-like phenotypes and proliferated well in response to CD3/CD28/CD2 stimulation, possibly as a compensatory mechanism for immune surveillance.

## Discussion

**Fig. 4:**
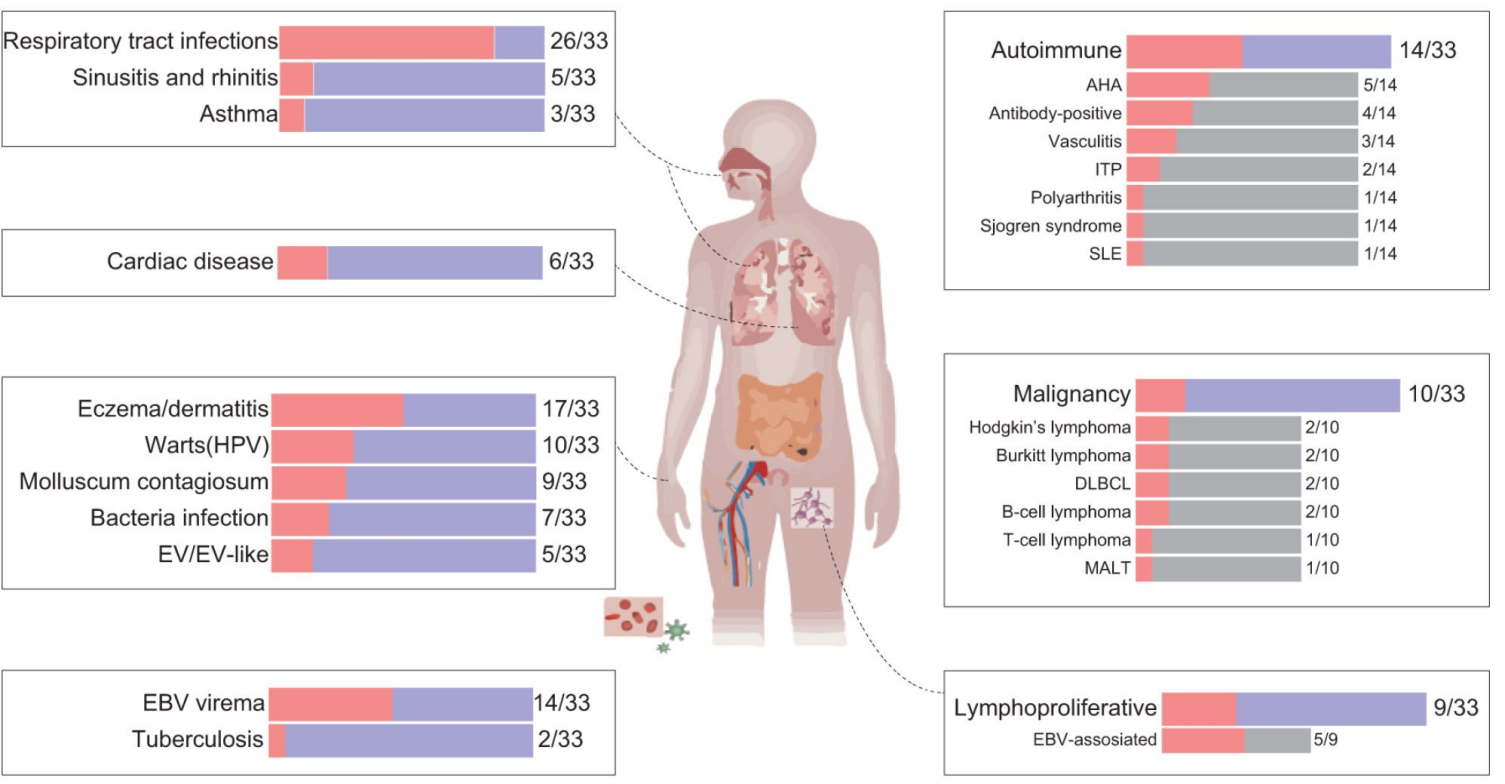
Schematic diagram of the clinical phenotype observed in 33 STK4-deficient patients. EV: Epidermodysplasia verruciformis, AHA: autoimmune hemolytic anemia, ITP: idiopathic thrombocytopenic purpura, SLE: systemic lupus erythematosus, DLBCL: diffuse large B-cell lymphoma, MALT: mucosa-associated lymphoid tissue

Lymphopenia, flat warts, atopic dermatitis, EBV viremia and lymphoma are common presentations in inherited or acquired immunodeficiencies. In this case, CD4^+^ T-cell lymphopenia prompted evaluation at an adult immunology clinic, and genetic testing revealed STK4 deficiency. STK4 deficiency can cause combined immune deficiency, but this patient had only mild skin phenotypes and EBV viremia, with no lymphoproliferative disorders or neutropenia. His immunoglobulin levels were normal, and he developed normal responses to vaccination against tetanus and diphtheria, indicating an absence of autoimmunity and no impairment of humoral immunity, consistent with the findings of normal B-cell levels in about two thirds of patients with STK4 deficiency (Table S3). The presence of DLBCL is unsurprising, as the relative risk of lymphoma is 8 to 10 times higher in patients with IEI than in immunocompetent individuals [34], with DLBCL diagnosed at earlier ages in IEI patients (41 vs. 55 years) [35]. Including this case, 33 patients with STK4 deficiency have been reported in total, 10 of whom had lymphoma (Fig. 4). Another nine cases developed non-malignant lymphoproliferative manifestations and five of these cases had documented EBV infection. Heart disease occurs in a small percentage (6/33) of patients [36] and EV was observed in five of the 33 STK4*-*deficient patients (Fig. 4). Our patient tested positive for HPV38, which is considered a low-risk type for cancer [37, 38]. Other cutaneous manifestations, including molluscum contagiosum (9/33), viral warts (10/33), and eczema-like dermatitis (17/33) are common in STK4-deficient patients.

Interest in γδ T cells is growing due to their potent anti-infection and antitumor activities and their role in immunodeficiencies [29]. An expansion of the γδ T-cell population has been reported in patients with atypical SCID, and has been shown to be associated with CMV infection and autoimmune cytopenia [29]. Fourteen of the 33 STK4-deficient patients developed EBV viremia (Fig. 4); Five of the seven patients tested displayed a 13–46% expansion of the γδ T-cell population relative to the normal level of 2–8% among PBMCs. The high levels of CD56 expression on the Vδ2 T cells suggest an activation of the effector machinery to protect the STK4-deficient host against EBV infection. Indeed, Vδ2 T cells have been shown to be cytotoxic to EBV target cells in vitro and in mouse models. Vδ2 T cells can efficiently kill EBV-transformed autologous lymphoblastoid B cells through γ/δ-TCR and NKG2D receptor triggering and Fas and TRAIL engagement [39], and exosomes derived from Vδ2 T cells can eliminate EBV-associated tumor cells [39–41], demonstrating the important function of Vδ2 T cells. Another condition associated with severe EBV disease, ITK deficiency, is characterized by Vδ2^−^ γδ T-cell expansion [13], suggesting that γδ T-cell expansion may not be δ2-specific in the setting of EBV viremia.

In conclusion, we report a patient with STK4 deficiency diagnosed in his thirties, with HPV38-associated EV, CD4^+^ lymphopenia, lymphoma and EBV viremia. The Vδ2 T-cell expansion observed in this patient may serve as a compensatory mechanism bolstering antiviral defenses.

## Electronic Supplementary Material

Below is the link to the electronic supplementary material.

Supplementary Material 1

Supplementary Material 2

## Data Availability

No datasets were generated or analysed during the current study.
