# Supplementary material for: Epidermodysplasia Verruciformis and Vδ2 γδ T-cell Expansion in STK4 Deficiency
Source: J Clin Immunol. 2024 Aug 7;44(8):172. doi: 10.1007/s10875-024-01780-z (PMC11306306; doi:10.1007/s10875-024-01780-z)
Supplement: Supplementary file 1 — Supplementary Material 1 [file 10875_2024_1780_MOESM1_ESM.docx]

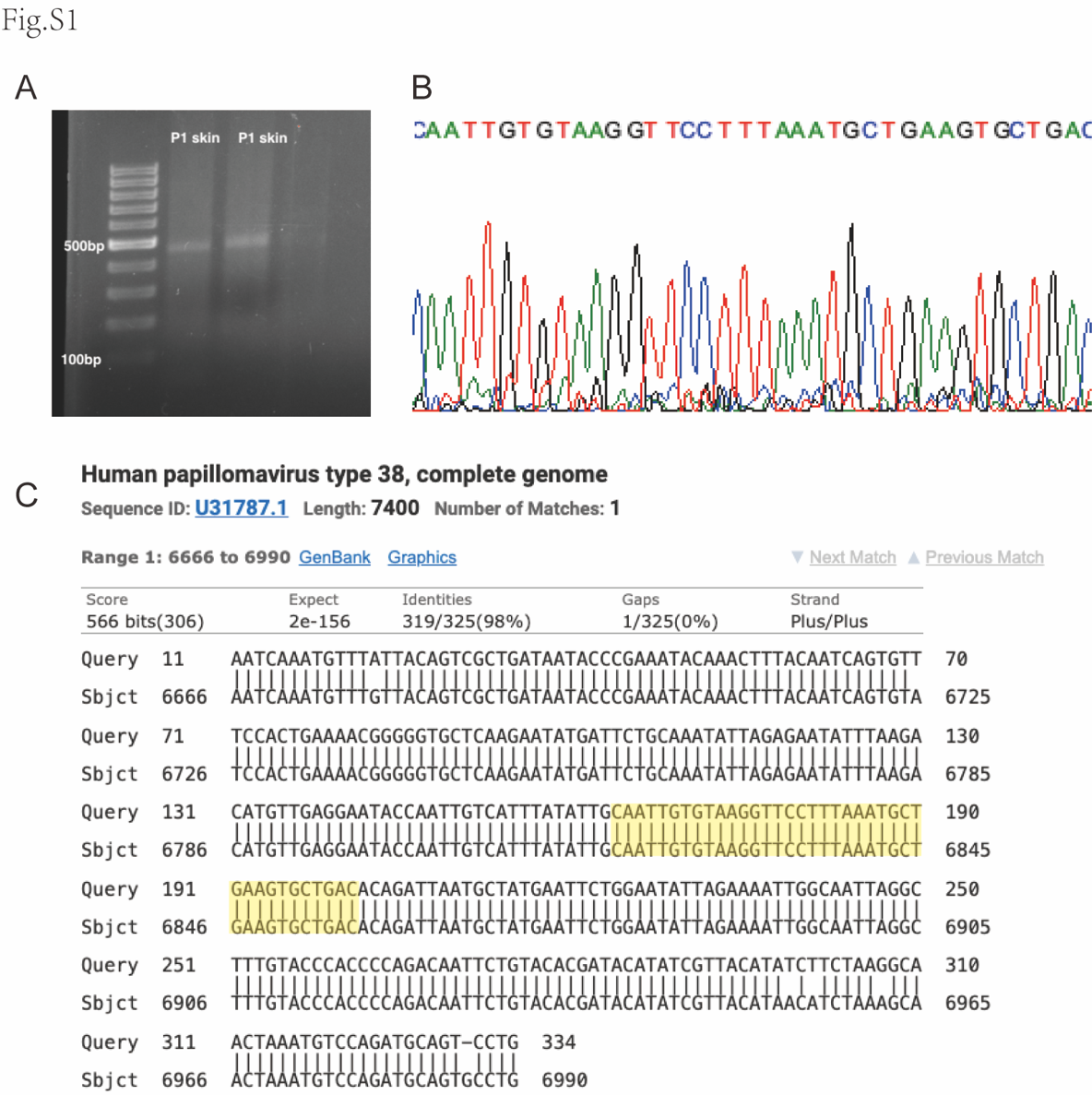
Figure S1 HPV genotyping in the proband with STK4 deficiency

A. PCR results for beta-HPV amplification from DNA isolated from a skin swab. B. Sanger sequence electropherogram. C. BLAST result indicating HPV38 infection.


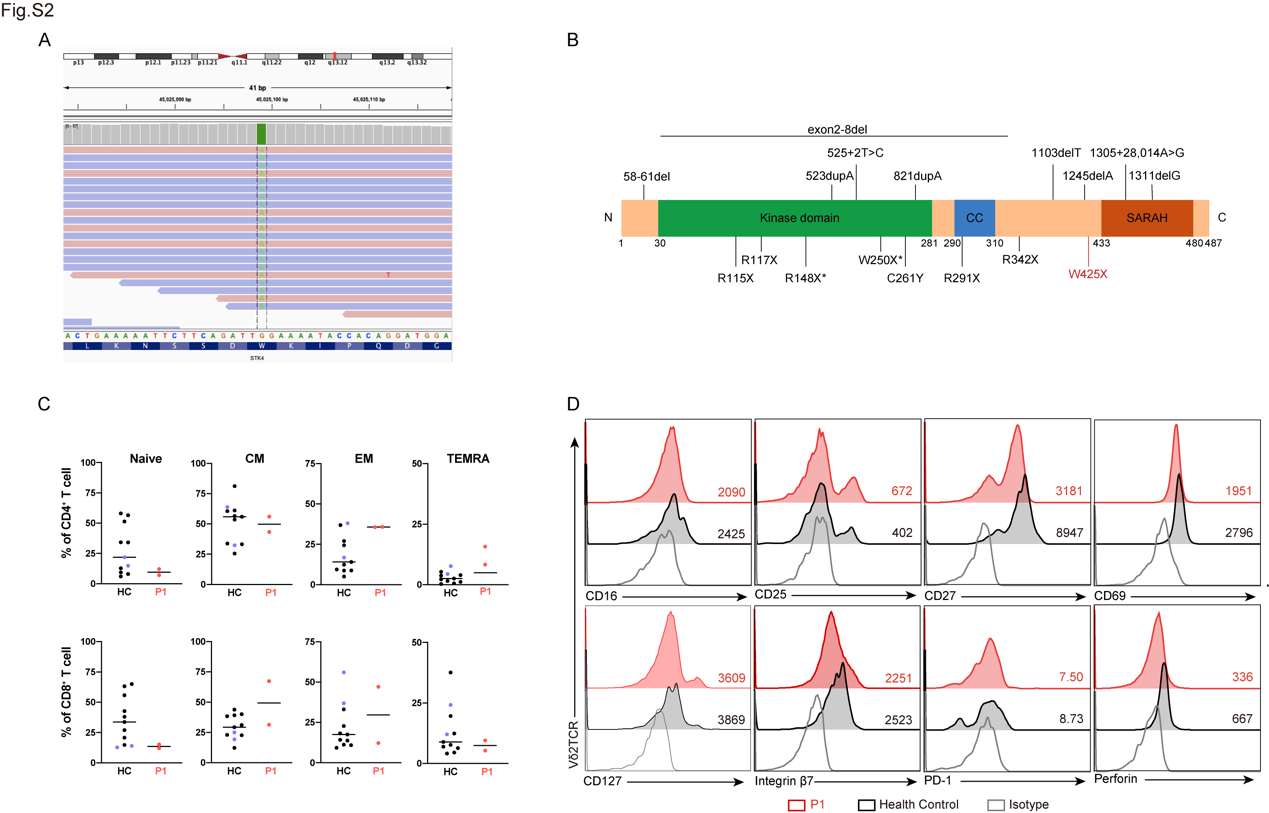


Figure S2 Pathogenic variants in STK4 deficiency and the immunophenotype of Vδ2 T cells

A.Integrative Genomics Viewer view of the variant from the WES data, which revealed the presence of a biallelic variant of the *STK4* gene (NM_006282.5:c.1274G>A, p.Trp425X); B.Schematic illustration of the STK4 protein with all the previously reported pathogenic variants. The variant identified in the patient is indicated in red. CC, coiled-coil domain; SARAH, Sav/Rassf/Hpo domain. *two different base mutations lead to the same amino-acid AA change. C. T-cell subsets identified on the basis of CD27 and CD45RA expression. Naïve T: CD45RA^+^CD27^+^, CM: CD45RA^-^CD27^+^, EM: CD45RA^-^CD27^-^, TEMRA: CD45RA^-^CD27^+^. D.CD16, CD25, CD27, CD69, CD127, PD-1 and integrin β7 expression in Vδ2 T cells.


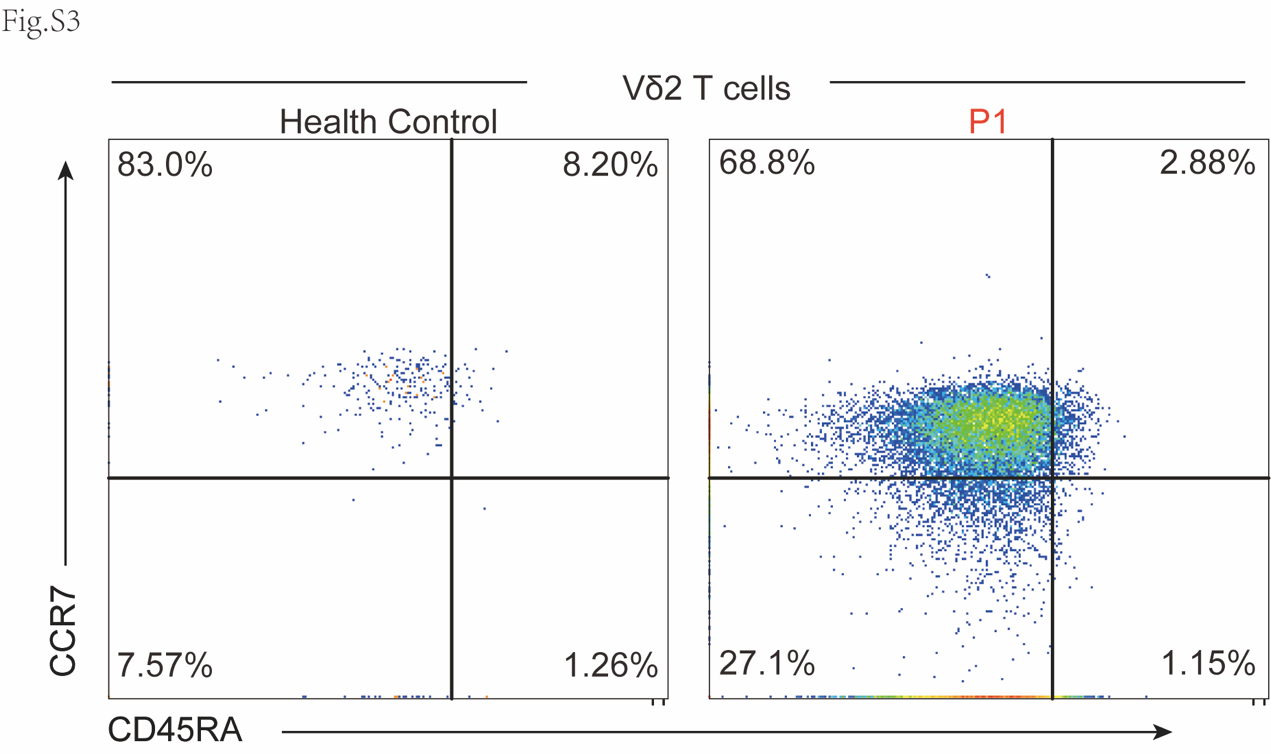
Figure S3 STK4-deficient patient displaying innate-like Vδ2^+^ cental memory T cells

CCR7^+^CD45RA^-^ T cells were the major subset of Vδ2 T cells.
